# Assessing Facial Thermal Nociceptive Response in Female Dogs After Elective Ovariohysterectomy Anesthetized with Isoflurane and Treated with Cannabidiol and Meloxicam Analgesia

**DOI:** 10.3390/ani15020227

**Published:** 2025-01-15

**Authors:** Alejandro Casas-Alvarado, Patricia Mora-Medina, Ismael Hernández-Avalos, Julio Martínez-Burnes, Agatha Miranda-Cortes, Adriana Domínguez-Oliva, Daniel Mota-Rojas

**Affiliations:** 1 PhD Program in Biological and Health Sciences [Doctorado en Ciencias Biológicas y de la Salud], Universidad Autónoma Metropolitana (UAM), Mexico City 04960, Mexico; 2 Facultad de Estudios Superiores Cuautitlán, Universidad Nacional Autónoma de México (UNAM), Cuautitlán 54714, Mexico; 3 Clinical Pharmacology and Veterinary Anesthesia, Biological Sciences Department, Facultad de Estudios Superiores Cuautitlán, Universidad Nacional Autónoma de México (UNAM), Cuautitlán 54714, Mexico; 4 Facultad de Medicina Veterinaria y Zootecnia, Instituto de Ecología Aplicada, Universidad Autónoma de Tamaulipas, Victoria City 87000, Mexico; 5 Neurophysiology of Pain, Behavior and Assessment of Welfare in Domestic Animals, Department of Animal Production and Agriculture, Universidad Autónoma Metropolitana (UAM), Mexico City 04960, Mexico

**Keywords:** dogs, pain, infrared thermography, cannabis, analgesia

## Abstract

Pain management requires the identification of certain indicators to recognize pain. Various tools have been suggested to achieve an objective evaluation, including infrared thermography (IRT). The objective of this study was to assess the facial thermal nociceptive response produced by the use of cannabidiol (CBD) alone and in combination with meloxicam in female dogs undergoing elective ovariohysterectomy anesthetized with isoflurane. Sixty-four female dogs of different breeds were randomly distributed into four study groups according to the treatment received. G1: Placebo group (*n =* 16); G2: Group receiving intravenous meloxicam as premedication (0.2 mg Kg^−1^) and every 24 h postoperatively 0.1 mg Kg^−1^ (*n =* 16); G3: Group treated with CBD (*n =* 16) at a dose of 2 mg kg^−1^ orally every 12 h; and G4: Group medicated with the combination of both treatments (*n =* 16). All treatments were administered for 48 h postoperatively. After the anesthetic surgical procedure, radiometric images were captured using IRT and physiological parameters during the events E_Basal_, E_30min_, E_1h_, E_2h_, E_3h_, E_4h_, E_8h_, E_12h_, E_24h_ and E_48h_. Overall, it was found that the high, medium and low temperatures of the thermal windows of the eye, upper eyelid and lower eyelid, as well as the average temperature of the lacrimal gland in G1 between events, were significantly lower at E_30min_, E_1h_ and E_2h_ compared to E_Basal_ (*p =* 0.01). Among treatments, a significantly higher temperature was observed in groups G2, G3 and G4 compared to G1 (*p =* 0.001) in the thermal windows of the upper eyelid, lower eyelid, lacrimal gland and ocular areas. Regarding physiological parameters, heart rate (HR) was higher in G1 compared to the animals in G2, G3 and G4 (*p =* 0.03). The respiratory rate (RR) was significantly lower in all four study groups during the postoperative events compared to their respective E_Basal_ (*p <* 0.05), while among treatments, G2, G3 and G4 had a lower RR compared to G1 (*p =* 0.03). Mild hypothermia was observed in all study groups at E_30min_ and E_1h_ compared to E_Basal_ (*p =* 0.001). No significant correlation was found between the temperatures of the assessed thermal regions and the physiological traits. In conclusion, CBD, whether administered alone or in combination with meloxicam, demonstrated comparable analgesic efficacy, which could control nociceptive cardiorespiratory and hemodynamic autonomic responses, as there were no significant changes in the facial thermal response between treatments G2, G3 and G4.

## Simple Summary

The precise assessment of pain remains a critical concern in ensuring the welfare of companion animals. Consequently, innovative technologies such as infrared thermography have been investigated. The aim of this study was to evaluate the facial thermal nociceptive response elicited by the administration of cannabidiol (CBD) alone and in combination with meloxicam in female dogs undergoing elective ovariohysterectomy under isoflurane anesthesia. Sixty-four bitches from different breeds were selected and randomly assigned into four groups according to the analgesic treatment. The groups were as follows: G1: Placebo group (*n =* 16). G2: Group premedicated with meloxicam at 0.2 mg kg^−1^ IV (*n =* 16). Postoperatively, this drug was used at 0.1 mg kg^−1^ every 24 h. G3: Group treated with CBD (*n =* 16) at a dose of 2 mg kg^−1^ orally every 12 h. G4: Group medicated with the combination of both treatments (*n =* 16). The findings revealed that the superficial temperature of the lacrimal gland, upper eyelid and lower eyelid was significantly lower in animals receiving analgesic treatments with meloxicam, CBD or a combination of the two when compared to the placebo group. This reduction correlated with physiological parameters such as heart rate and respiratory rate. Therefore, based on the superficial thermal response, it can be concluded that the use of CBD, either alone or in combination with meloxicam, exhibited comparable analgesic efficacy, effectively modulating nociceptive cardiorespiratory and hemodynamic autonomic responses, with no significant differences in facial thermal patterns observed between treatments.

## 1. Introduction

Acute pain in animals has gained relevance in recent years due to its negative impact from a hemodynamic, immunological, behavioral and sensory perspective [1,2]. Therefore, its recognition, categorization and treatment are essential for safeguarding animal welfare [3,4]. Despite the importance of this task, various factors have been reported that hinder its treatment, some related to the patient, such as species, breed and age, and others inherent to the observer, such as the sex and age of the evaluator, experience in handling different evaluation scales and even knowledge of these scales [5,6,7].

The application of technology for recognizing acute pain has been proposed, such as infrared thermography (IRT), which allows for the evaluation of superficial microvascular temperature. IRT uses a camera with specialized lenses to detect infrared radiation. Thus, it has been suggested as a non-invasive method that can evaluate changes in surface temperature in regions with high amounts of blood capillaries associated with sympathetic fibers. In this way, the surface temperature of an individual, which is modified by the dermal microcirculation, can be remotely assessed through IRT [8]. Thus, IRT has been suggested as a non-invasive technique to evaluate noxious events that cause pain and inflammation, which is accompanied by a significant increase in temperature [9]. During these events, the activation of the autonomic system (ANS) and the release of catecholamines cause vasoconstriction and a decrease in skin temperature, which is detected by IRT and can objectively recognize autonomic activity due to acute pain perception. Therefore, the changes in the surface blood circulation detected with IRT can be associated with the animal’s thermal stability under different conditions, not only pain [8,10,11,12,13,14] but also for recognizing periods of fever due to infectious states [15] or evaluating thermal stress in animals [16]. However, information on dogs is still scarce, especially reports suggesting the decrease in surface temperature due to the activation of the Sympathetic Nervous System (SNS) and the perception of acute pain, as observed in other species [17,18,19].

The implementation of IRT in the study and monitoring of pain in animals has led to the possibility of developing and applying this tool in the validation of the analgesic efficacy and clinical safety of some drugs commonly used during anesthetic surgical procedures [12,20]. In this regard, it is necessary to point out that compassionate care and pharmacological intervention for pain have had significant updates, as new drugs, such as phytocannabinoids, have been suggested and shown to reduce pain perception similarly to conventional analgesics [21,22,23,24,25,26]. However, the evidence regarding the use of these drugs for the control of acute postoperative pain is still limited. Therefore, the objective of the present study was to evaluate the facial thermal nociceptive response to the use of cannabidiol (CBD) alone and in combination with meloxicam in female dogs following elective ovariohysterectomy during the postoperative period under the hypothesis that the use of CBD will generate analgesic control similar to meloxicam, an effect that will be reflected in a stable cardiorespiratory and hemodynamic autonomic response to a perioperative nociceptive stimulus.

## 2. Materials and Methods

### 2.1. Animals

This study evaluated 64 female dogs of different breeds, with an average age of 2 ± 1.5 years, a body condition score of 4/9 according to the body condition score system [27] and an average weight of 12.1 ± 2.3 kg. The sample size was estimated using G*power 3.1.9.7 software (Heinrich-Heine-Universität Düsseldorf, Düsseldorf, Germany). It was determined that the total sample size was 64 animals, considering an alpha error probability (α) of 0.05, a confidence level of 95%, a power (probability of error 1 − α) of 0.95 and a correlation between repeated measures of 0.5, for four experimental groups with ten measurements [28].

All admitted animals underwent a complete general physical examination, blood cell count, serum biochemistry and urinalysis to select healthy animals or those with an ASA-1 anesthetic risk, according to the American Society of Anesthesiologists [29]. Animals presenting any condition that would cause acute pain, with an ASA-3 risk or higher, and those with any severe infectious disease were excluded.

### 2.2. Experimental Desing

The experimental study had a prospective, blind and randomized design. The 64 animals were randomly assigned to four groups according to the treatment administered. G1 (*n =* 16) was the placebo group medicated with 1 mL of 0.9% sodium chloride intravenously (IV); G2 (*n =* 16) received CBD at a dose of 2 mg kg^−1^ orally (PO) 30 min prior to induction [22]; G3 (*n =* 16) was medicated with meloxicam (meloxivet, Norvet, Mexico city, Mexico) at a dose of 0.2 mg kg^−1^ IV 30 min prior to induction and in G4 (*n =* 16), CBD at 2 mg kg^−1^ PO and 10 min after, meloxicam was administered at 0.2 mg kg^−1^ IV [30]. From the first administration and for 48 h post-surgery, CBD was administered every 12 h, and in the case of meloxicam, the dose was reduced to 0.1 mg kg^−1^ every 24 h.

The assessment of IRT variables and physiological parameters was conducted at the following time points: Baseline, considered 1 h before premedication (E_Basal_), 30 min post-surgery (E_30min_), 1 h post-surgery (E_1h_), 2 h post-surgery (E_2h_), 3 h post-surgery (E_3h_), 4 h post-surgery (E_4h_), 8 h post-surgery (E_8h_), 12 h post-surgery (E_12h_), 24 h post-surgery (E_24h_) and 48 h post-surgery (E_48h_).

### 2.3. Anesthetic–Surgical Management

Surgical procedures were elective and performed following written informed consent from the owners. Study subjects underwent a 4 h water fast and a 6 h food fast prior to anesthesia. Aseptic catheterization of the cephalic vein was performed using a 20 G catheter, through which lactated Ringer’s solution was administered intravenously (IV) at a rate of 5 mL kg^−1^ h^−1^ (BeneFusion VP1 Vet, Mindray, Darmstadt, Germany) during the surgical procedure [31].

After catheterization, Dexmedetomidine (Dexdomitor, Zoetis, Mexico City, Mexico) was administered intravenously at a dose of 1.5 µg kg^−1^. The dogs exhibited moderate sedation, which was assessed 5 min after sedative administration using the sedation scale proposed by Grint et al. [32]. Anesthetic induction was achieved with Propofol (Recofol, Pisa, Mexico City, Mexico) at 2–4 mg kg^−1^ IV [33]. Orotracheal intubation was performed upon observation of ventromedial deviation of the eyeball and decreased mandibular tone. Subsequently, patients were connected to a rebreathing anesthetic circuit for administration of 100% oxygen at a flow rate of 45 mL kg^−1^ min^−1^. Anesthesia maintenance was achieved through the vaporization of isoflurane (Sofloran, Pisa, Mexico City, Mexico) with the dial set to 1.7%, adjusting this concentration according to the required anesthetic depth to maintain a mean arterial pressure (MAP) of 60–90 mmHg. The depth of surgical anesthesia was assessed through clinical indicators, including mandibular muscle relaxation, the eyeball’s ventromedial rotation and the palpebral reflex’s absence. Throughout the anesthesia–surgical procedure, all animals were mechanically ventilated in pressure-controlled mode with a peak inspiratory pressure (Paw) of 12–15 cmH_2_O, an inspiratory-to-expiratory ratio (I:E) of 1:2, a respiratory rate (RR) of 12–15 breaths per minute and an inspiratory pause time (Tramp) of 0.6 s, using a mechanical ventilator integrated into the anesthesia station (Wato-EX20 vet, Mindray, Darmstadt, Germany), with the settings adjusted to maintain an end-tidal carbon dioxide (ETCO_2_) level of 35–45 mmHg.

The same anesthesiologist and surgeon consistently carried out all anesthesia and surgical procedures.

### 2.4. Infrared Thermography

The radiometric images were captured using a thermal camera (FLIR E86, Wilsonville, OR, USA). All images were acquired at a distance of 1 m from the facial region with an emissivity of 0.95. Subsequently, these images were downloaded and processed using FLIR Tools software (Ver. 6.4.18039.1003) to delineate thermal windows.

**Figure 1:**
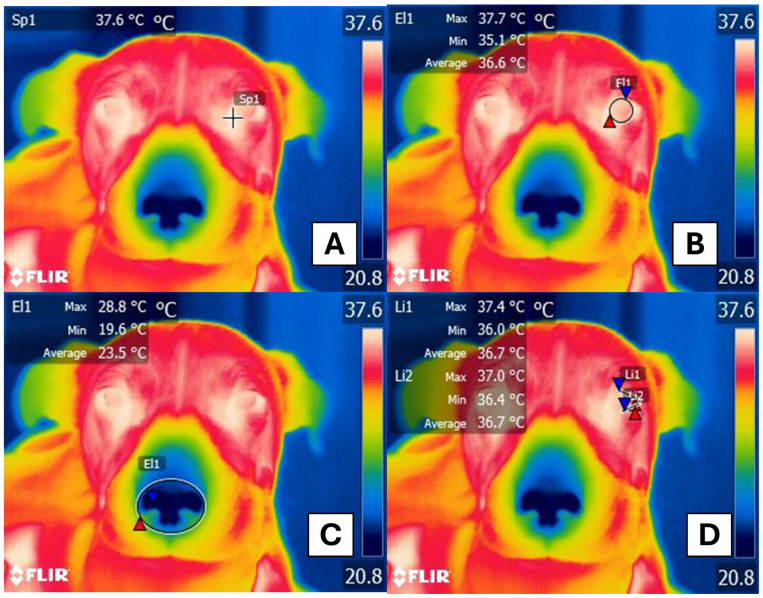
Thermal windows traced on radiometric images in female dogs. (**A**). Lacrimal gland window. A focal point (Sp1) was used at the medial canthus of the eyelids. (**B**). Ocular thermal window. A circle of approximately 2 cm in diameter (El1) was used to encompass both the upper and lower eyelids. (**C**). Nasal thermal window. A circle of 2.5 cm in diameter (El1) was used to encompass both nostrils. (**D**). Eyelid thermal window. A line of 4 cm in length was used to encompass the outer corner of both the lower eyelid (Li2) and the upper eyelid (Li1). Red triangles: maximum temperatures; blue triangles: minimum temperatures.

The thermal windows of interest were manually delimited on the image. For the left upper and lower eyelids, a line of approximately 4 cm in length was drawn, while a circle with a diameter of 2 cm was used for the ocular window on the left eye. Additionally, an ellipse with a diameter of 2.5 cm was used for the nasal window to obtain high, medium and low temperatures, as depicted in Figure 1. Lastly, a focal point was signaled at 1 mm from the medial canthus of the left eye’s eyelids to acquire only the average temperature for the lacrimal gland window [11].

### 2.5. Monitoring and Recording of Physiological Parameters

These variables were recorded by a single blinded veterinarian, who had undergone prior training throughout all evaluation events. Heart rate (HR) was assessed through direct auscultation of cardiac sounds using a flexible stethoscope (Littmann, 3M, Saint Paul, MN, USA) over the left intercostal regions 3, 4 and 5 for 1 min. The respiratory rate (RR) was evaluated by observing chest inspiratory movements for 1 min. Finally, body temperature (T °C) was recorded using a digital clinical thermometer via rectal route (Neutek, Mexico).

### 2.6. Post-Surgical Rescue Analgesia Protocol

If animals obtained a score of ≥6 on the Glasgow pain scale, tramadol (2 mg Kg^−1^ IV) was administered as rescue analgesia [34].

### 2.7. Statical Analysis

Descriptive statistics were obtained using the statistical package GraphPad Prism (ver. 10.0.2) for all groups (G1, G2, G3 and G4) and each of the study events (E_Basal_, E_30min_, E_1h_, E_2h_, E_3h_, E_4h_, E_8h_, E_12h_, E_24h_ and E_48h_). The Kolmogorov–Smirnov test was employed to assess data normality for all evaluated variables.

Treatments were considered independent variables, while each of the thermal windows and physiological parameters were regarded as dependent variables. To evaluate the effects of these variables, a mixed linear model was employed using the following framework:

Yijak = µ + τi + τj + τiτj + βk + eij

where

Y = Response variable (IRT, physiological parameters).

τi = Treatment effect (G1, G2, G3 and G4).

τj = Event effect (E_Basal_, E_30min_, E_1h_, E_2h_, E_3h_, E_4h_, E_8h_, E_12h_, E_24h_ and E_48h_).

β = Random effect (animal).

µ = Population mean.

e = Residual.

To assess differences between means, Tukey’s post hoc test was employed. A significance level of *p* < 0.05 was set for all cases. Correlations between variables were determined using Pearson’s correlation.

### 2.8. Ethical Statement

All procedures strictly followed the guidelines set forth in Mexico’s Official Norm NOM-062-ZOO-1999 [35], which provides technical specifications for the care, management and ethical treatment of animals in ethological research. The project received approval from the Academic Committee of the Ph.D. Program in Biological and Health Sciences (registration number CBS.066.21). Additionally, the study adhered to the ARRIVE guidelines and maintained the highest ethical standards for animal experimentation [36,37]. Importantly, no surgical procedure or data collection step caused harm, mutilation or undue distress to the animals, ensuring their ethical treatment throughout the study. Prior to the study, informed consent was obtained from the animals’ owners, permitting the procedures.

## 3. Results

**Figure 2:**
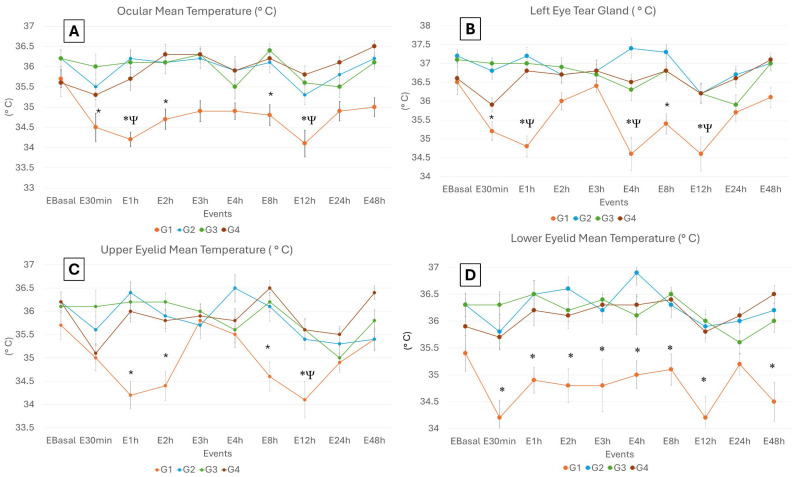
Temperatures (mean ± SE) of the four thermal windows at the evaluation events (E) of 64 female dogs undergoing elective ovariohysterectomy distributed into 4 study groups: G1, G2, G3 and G4. (**A**). Mean ocular temperature at E_30min_, E_1h_, E_2h_, E_8h_ and E_12h_; the surface temperature of G2, G3 and G4 was significantly higher than G1. (**B**). Left eye tear gland. It can be observed that the surface temperature of G2, G3 and G4 was significantly higher at E_1h_, E_4h_, E_8h_ and E_12h_ than G1; however, G1 had significantly lower temperatures at E_1h_, E_8h_ and E_12h_ in contrast to E_Basal_. (**C**). Upper eyelid mean temperature. The surface temperature of G2, G3 and G4 was significantly higher at E_1h_, E_2h_, E_8h_ and E_12h_ than in G1, while G1 had lower temperatures at E_12h_ than at E_Basal_. (**D**). Lower eyelid mean temperature. A significantly higher surface temperature was recorded in G2, G3 and G4 at E_30min_, E_1h_, E_2h_, E_3h_, E_4h_, E_8h_, E_12h_ and E_24h_ when compared to G1. * Indicates significant differences between treatments of the same event (*p <* 0.05), and Ψ indicates significant differences between events for the same treatment (*p* < 0.05).

Overall, it was found that the surface temperature of the different thermal windows was significantly higher in animals belonging to G2, G3 and G4 compared to G1 (*p* < 0.05), as shown in Table 1. Specifically, in the case of high ocular temperature, a significant decrease of 1.9 °C was observed in G1 compared to G2, G3 and G4 at E_2h_ (*p =* 0.0005). Meanwhile, for the variable of medium ocular temperature (Figure 2), a significant decrease of 1.5 °C and 1.6 °C was observed at E_1h_ and E_12h_ compared to E_Basal_ in G1 (*p =* 0.03). In this same variable, between treatments, the surface temperature was 2 °C lower in G1 compared to G2, G3 and G4 at E_1h_ (*p =* 0.0001). However, at E_30min_ and E_8h_, it was observed that the temperature in G3 was 1.5 °C (*p =* 0.03) and 1.6 °C (*p =* 0.01) higher compared to G1, while at E_2h_ and E_12h_, the temperature in G4 was 1.6 °C (*p =* 0.02) and 1.7 °C (*p =* 0.01) higher compared to G1. Regarding low ocular temperature, it was only found that at E_30min_, the surface temperature of G1 was 2.8 °C lower compared to G2, G3 and G4 (*p =* 0.05). Similarly, the temperature of G1 showed a significant decrease of 1.9 °C and 3.7 °C compared to animals receiving analgesic treatment (G2, G3 and G4) at E_2h_ (*p =* 0.03) and E_3h_ (*p =* 0.006), respectively.

Regarding the temperature observed in the lacrimal gland (Figure 2), differences were also noted among the evaluation events, where the surface temperature at E_Basal_, E_2h_, E_3h_ and E_48h_ was significantly 1.5 °C higher compared to E_1h_, E_4h_ and E_12h_ in G1 (*p* = 0.001). Similarly, between treatments, it was found that the surface temperature of G2 and G3 was 1.8 °C and 1.6 °C higher, respectively, compared to G1 at E_30min_ (*p =* 0.01). On the other hand, in the same variable, the temperature of G1 was significantly 2 °C lower compared to G2, G3 and G4 at E_1h_ (*p =* 0.0001). Similarly, at E_4h_ and E_12h_, it was observed that the temperature of G1 was significantly 2.8 °C (*p =* 0.0005) and 1.6 °C *(p =* 0.004) lower compared to G2, G3 and G4. Meanwhile, at E_8h_, the temperature of G3 was significantly 1.9 °C higher compared to G1 (*p =* 0.001).

The upper eyelid’s high temperature was significantly 1.3 °C lower at E_30min_ and E_1hr_ compared to E_2h_, E_4h_, E_8h_ and E_48h_ (*p =* 0.006) in G1. Likewise, it was found that at E_12h_, E_24h_ and E_48h_, the temperature was between 0.7 and 1.5 °C, which is significantly lower compared to the rest of the evaluation events in G3 (*p =* 0.02). Moreover, in this same variable, it was found that the temperature in G3 was significantly 1.7 °C higher compared to G1 at E_30min_ (*p =* 0.0006). Meanwhile, at E_1h_, the temperature presented by G2, G3 and G4 was 1.7 °C higher compared to G1 (*p =* 0.002). Regarding the mean temperature of the upper eyelid (Figure 2), it was found that the temperature at E_30min_ and E_8h_ was significantly 1.6 °C and 1.5 °C lower compared to E_Basal_ in G1 (*p =* 0.03). However, in the G4 events, the temperature at E_30min_ was only found to be 1.4 °C lower compared to E_8h_ (*p =* 0.03). Among treatments, the mean temperatures of G2, G3 and G4 were 2.2 °C, 2 °C and 1.8 °C, respectively, which were significantly higher when compared with G1 in E_1h_ (*p =* 0.009). At E_2h,_ the temperatures of G2, G3 and G4 were 1.5 °C, 1.8 °C and 1.4 °C, respectively, higher than G1 (*p =* 0.0006). At E_8h_, the temperatures of G2, G3 and G4 were higher by 1.5 °C, 1.6 °C and 1.9 °C, respectively, compared with G1 (*p =* 0.001). Similarly, at E_12h_, G2, G3 and G4 recorded higher temperatures by up to 1.3 °C, 1.5 °C and 0.6 °C, respectively, when compared with G1 (*p =* 0.01). In the variable of upper eyelid low temperature, it was observed that among events, the E_Basal_ temperature was between 0.6 °C and 2.9 °C higher compared to the rest of the evaluation of the post-surgical events in G1 (*p =* 0.01). Among treatments, it was observed that the temperature of G1 was 3.3 °C (*p =* 0.0001), 1.7 °C (*p =* 0.05), 2.5 °C (*p =* 0.003), 3.3 °C (*p =* 0.003) and 2.9 °C (*p =* 0.01) lower compared to G2, G3 and G4 at E_30min_, E_1h_, E_2h_, E_3h_ and E_12h_, respectively. At E_4h_, it was observed that the temperature in G2 and G4 was 1.4 °C higher compared to G1 (*p =* 0.05).

Regarding the high temperature of the lower eyelid among events, it was observed that at E_30min_, E_1h_, E_2h_ and E_12h_, the temperature was 1.5 °C lower compared to E_Basal_ in G1 (*p =* 0.05). Among treatments, it was observed that G1 exhibited a temperature of 2 °C less compared to G2, G3 and G4 at E_Basal_ (*p =* 0.004). Meanwhile, at E_1h_, G2 and G3 had a temperature 0.9 °C higher compared to G4 and 1.5 °C higher compared to G1 (*p =* 0.05). In the mean temperature of the lower eyelid (Figure 2), it was observed that among treatments, the temperature of G2, G3 and G4 was 1.6–2 °C higher compared to G1 at E_1h_ (*p =* 0.01), E_2h_ (*p =* 0.02), E_3h_ (*p =* 0.002), E_12h_ (*p =* 0.01) and E_48h_ (*p =* 0.0008). In the case of E_4h_, in G2, the temperature was 1.9 °C higher compared to G1 (*p =* 0.0003). For the low temperature of the lower eyelid, it was observed that G2, G3 and G4 were between 2.5 °C and 1.9 °C significantly higher compared to G1 at E_30min_ (*p =* 0.004), E_2h_ (*p =* 0.02), E_3h_ (*p =* 0.05), E_8h_ (*p =* 0.03), E_12h_ (*p =* 0.01) and E_48h_ (*p =* 0.009). At E_1h_, G2 and G3 had a temperature of 2.5 °C and 2.3 °C higher than G1 (*p =* 0.002), while G2 had a temperature 2.3 °C higher compared to G1 at E_4h_ (*p =* 0.01). Regarding the high nasal temperature variable, in G4, at E_12h_, the temperature was 3 °C compared to E_Basal_, E_2h_, E_3h_, E_4h_ and E_8h_ (*p =* 0.05).

On the other hand, Table 2 presents the values of the physiological indicators where differences were observed between events in G2, with an increase in HR at E_Basal_ of 20 beats per minute (bpm) compared to the rest of the events (*p =* 0.02). In the case of differences between treatments, it was observed that G2, G3 and G4 presented a lower HR—by 9 to 15 bpm—than G1 at E_30min_ (*p =* 0.05). At E_12h_, G2, G3 and G4 had a lower HR than G1 (16 bpm) (*p =* 0.05). For RR, it was observed that the breaths per minute (bpm) were between 4 and 8 lower in the rest of the events compared to E_Basal_ in G1 and G2 (*p =* 0.03). Likewise, in G3 and G4, all post-surgical events showed results between 3 and 10 bpm lower compared to E_Basal_ (*p =* 0.04). As for differences between treatments, it was found that G1 had 10 bpm less than G2, G3 and G4 at E_1h_ (*p =* 0.05), E_2h_ (*p =* 0.02) and E_8h_ (*p =* 0.01). In the case of E_30min_, it was observed that G2 had 4 bpm higher results compared to G4 and 5 bpm in G1, while G3 had results 11 bpm lower compared to the rest of the treatments (*p =* 0.05). The temperature (°C) showed differences between events; for G1, at E_30min_, the temperature was 1.4 °C lower than E_Basal_ and E_24h_ (*p =* 0.006). For G2, at E_30min_, the temperature was lower compared to the rest of the post-surgical events (*p =* 0.007). In G3, it was observed that the temperature at E_30min_ was 1 °C lower than E_Basal_ (*p =* 0.001). For G4, at E_30min_ and E_1h_, the temperature was 1.1 °C and 0.9 °C lower compared to E_Basal_ (*p =* 0.01).

There were no correlations between the surface temperature obtained in the thermal windows and the physiological parameters, where no correlation was observed between the physiological parameters and the different thermal windows of the upper eyelid, ocular, lacrimal gland, lower eyelid and nasal area (Table 3). However, significant positive correlations (*p =* 0.001) were observed among the thermal windows.

## 4. Discussion

Overall, the results of the present study indicate that in G1 (placebo group), the surface temperature in all thermal windows was lower compared to animals receiving analgesic treatment (*p <* 0.05). Acute pain activates the hypothalamic–pituitary–adrenal (HPA) axis, which causes peripheral vasoconstriction phenomena and decreases microcirculation in blood capillaries closer to the dermal surface [38,39]. This phenomenon has also been observed in an experimental model of bovines under surgical conditions, where the perception of acute pain may induce the activation of the (HPA) axis, which can also modulate the release of catecholamines in the adrenal medulla, thus modifying the subsequent surface thermal response to a surgical painful stimulus [17,40,41,42]. Thus, this could reinforce the idea that IRT may be a tool associated with the activity of the ANS [43,44].

The findings are similar to what was observed in the physiological parameters. For example, HR of G2, G3 and G4 presented a significantly lower bpm compared to G1 at E_1h_ (*p =* 0.05) and E_2h_ (*p =* 0.05). Changes in this physiological parameter would be explained by the general effect of pain. Painful stimuli are processed by the peripheral nociceptors and transmitted by these same fibers to the dorsal horn of the spinal cord. Subsequently, the signal is projected through the spinothalamic and spinoreticular tracts to brain regions, such as the somatosensory cortex that can intercommunicate with the thalamus and hypothalamus [45,46]. In general, the Central Nervous System (CNS) confers the characteristics of intensity and direction but also provides a behavioral and physiological response to pain. Additionally, the activation of the HPAaxis leads to the modification of physiological parameters such as HR and blood pressure [47]. According to authors such as Mansour et al. [2] and Hernández-Avalos et al. [48], pain can increase HR and blood pressure by up to 20% with respect to its basal parameter. Therefore, the groups that received meloxicam (G2), CBD (G3) or the combination of both (G4) had a lower RR than the placebo group (G1), which might be related to lower pain perception. This would explain the differences found in the RR between G2, G3 and G4 compared to G1 at E_30min_, E_1h_, E_2h_ and E_8h_. As an additional explanation, the increase in physiological parameters can be attributed to the activation of the ANS when perceiving a potentially challenging event, facilitating the availability of energy resources [49,50]. When an animal perceives acute pain, the activation of the ANS releases catecholamines, which influence the physiological parameters assessed in the present study, including the thermal response. Thus, IRT would help to evaluate autonomic activation indirectly.

Similarly, it has been documented that the anatomical regions used in the present study have specific characteristics that can help recognize the ANS activity. For example, it has been mentioned in both dogs and ruminants at the level of the lacrimal caruncle that this anatomical region is vascularized by the infraorbital artery, which is innervated by sympathetic fibers from the facial nerve [11]. Thus, in potentially stressful events such as the perception of acute pain, it is understood that during the E_30min_ and E_1h_, a decrease in radiated temperature was observed in the G1 group when assessing the ocular maximum temperature, the upper eyelid maximum temperature and the lower eyelid maximum, average and minimum temperature (*p <* 0.05), which possibly suggests that these were the most painful post-surgical events the animals experienced. In the ocular region, eyelids and lacrimal gland, local vascularization is provided by the infraorbital, supraorbital and maxillary arteries. These facial regions are also innervated by sympathetic fibers from the facial nerve that, by increasing the sympathetic activity of the ANS, release neurotransmitters such as catecholamines. Consequently, this decreases heat exchange with the environment, and this is reflected in a lower surface temperature [8,12]. Therefore, this explanation would suggest the application of IRT as an indirect method of evaluating the ANS to recognize acute pain in this species during the immediate postoperative period.

The analgesic activity of CBD uses two main mechanisms of action. On the first hand, it is attributed to the presence of CB1 receptors in the CNS, such as the cerebral cortex, spinal cord, periaqueductal gray matter and cerebellum [51,52,53], as well as CB2 receptors in cells of the immune system and visceral tissue [54,55,56,57]. The agonism of CB1 receptors inhibits the activity of the Gi/o protein, leading to the inhibition of adenylyl cyclase activity and reduced cAMP synthesis. Additionally, it generates voltage-dependent calcium channel blockade and increased potassium conductance [23,58,59]. Collectively, these mechanisms result in reduced presynaptic neurotransmitter release, including catecholamines, histamine, serotonin, dopamine, cholecystokinin and glutamate in the CNS, thereby diminishing nociceptive stimuli in central regions. On the other hand, the agonism of CBD to CB2 receptors can lead to decreased inflammatory response during surgical stimulation, involving tumor necrosis factor-α (TNF-α) and interleukins released from microglia or macrophages [60,61]. CBD usage has been observed to reduce pro-inflammatory factors such as interleukin (IL)-10, IL-1, IL-8, nuclear factor-κB and TNF-α due to decreased cyclooxygenase-2 (COX-2) expression [62,63]. By controlling the inflammatory process, nociceptive transmission and transduction may be reduced [47,64]. Thus, the control of the SNS activity due to the analgesic effect of CBD might be related to the findings of the present study. The significant increases in the surface temperature of the ocular region, eyelids and lacrimal caruncle in G3 and G4, when compared with G1 (*p <* 0.05), could be associated with the analgesic effect presented by this treatment. Likewise, in the same group, the HR and RR were significantly lower compared to G1 (*p <* 0.05). These findings suggest that CBD can manage pain-related physiological changes, presenting the first report on the analgesic effect of CBD to control post-surgical pain, posing it as an alternative clinical application of this treatment [21].

In G2, it was observed that the surface temperature was significantly higher than G1 (*p <* 0.05). This could be related to the action mechanism of meloxicam, which preferentially inhibits COX-2, actively involved in inflammatory processes, leading to prostaglandin synthesis inhibition and inhibition of the transduction and transmission of noxious stimuli, as well as the expression of phospholipase A2, substance *p*, serotonin, histamine, PGE2 and pro-inflammatory cytokines in neurons of the dorsal horn of the spinal cord [65,66,67]. Meloxicam does not only control inflammation but can reduce acute pain due to the synthesis inhibition of pro-inflammatory substances such as PGE2 and cytokines, which participate in the transmission and processing of the nociceptive stimulus [68]. Consequently, meloxicam can prevent the formation of substances promoting nociceptive stimuli and the occurrence of peripheral and central sensitization phenomena [69,70]. This analgesic effect would also explain the progressive decrease in HR in G2 (*p <* 0.05), as analgesics provide cardiovascular and ventilatory stability by avoiding the increase in these physiological parameters. This suggests that an analgesic protocol can reduce the need for general anesthetics and might be related to the significant decrease in HR in post-surgical E compared to E_Basal_ in G2 (*p* < 0.05). Moreover, these medications are recommended to control surgical stress during anesthesia [71,72,73]. This perspective could also explain the effect observed with the progressive decrease in surface temperature in G1, G2, G3 and G4 (*p* < 0.05) since the use of general anesthetics inhibits temperature control by decreasing the response at the hypothalamic level and increases heat loss due to vasodilation of superficial capillaries [44]. Thus, it is imperative to provide preventive pain management to surgical patients.

The combined effect of both treatments can be observed in the results of G4, where a general and significantly lower surface temperature was recorded in the ocular, eyelid and lacrimal caruncle thermal windows compared to G1 (*p* < 0.05). The combination of analgesics of different classes allows pain management by inhibiting different pathways of the neurobiology of pain [64,74]. Since both treatments share the same mechanism of action, it could improve the analgesic effectiveness; however, this needs further research because there were no differences between G2, G3 and G4 (*p >* 0.05). In summary, the pharmacological basis of both drugs would explain why the surface temperature of G2 and G3 was significantly lower compared to G1 (*p <* 0.05) and why HR and RR were significantly lower in G2 and G3 compared to G1 at E_1h_ and E_2h_ (*p <* 0.05). Therefore, based on our findings, it is feasible to argue that using CBD, meloxicam or their combination allows for controlling the SNSi tone and, consequently, autonomic hemodynamic activity [75]. This aligns with previous findings by Stewart et al. [17], who observed that using local analgesics in a bovine model controlled SNS activity in animals undergoing dehorning surgery. Similarly, in dogs, it has been mentioned that this phenomenon can be managed using analgesics such as meloxicam or opioids [2,48]. Thus, the control of this response, as observed in this study, may serve as clear evidence of the analgesic activity of CBD following a surgical stimulus, thus supporting its implementation as an intraoperative analgesic treatment.

On the other hand, a null correlation between thermal windows and physiological parameters was observed (r = 0.01, *p >* 0.05), Johnson [76] mentions that the poor correlation of IRT with other physiological indicators might be due to acute pain processing. Pain is integrated into CNS regions such as the cerebral cortex, hypothalamus, thalamus and amygdala. However, this does not occur in all cases because the control of the vasomotor response or the pupillary response can often be regulated locally and does not necessarily involve the CNS. The positive correlation found between the thermal windows (*r =* 0.45*–*0.86, *p <* 0.0001) might be associated with the thermoregulatory mechanisms. When an organism is exposed to heat or cold stimulus, the lateral parabrachial nucleus of the hypothalamus activates neurons of the dorsal subnucleus and projects the information to the median preoptic nucleus where the information is modulated in the rostral magnus raphe to later transmit this signal to the sympathetic preganglionic fibers. These fibers innervate the cutaneous blood vessels where vasodilation or vasoconstriction is promoted depending on the case [77,78,79]. This would explain that this response generally occurs in all thermal windows and its positive correlation between them. Moreover, although the present study found significant differences in the physiological parameters, the small changes in some of these might be related to other factors, such as stress, the environment or the observer’s presence, which might influence the thermal response [80,81]. Due to the lack of a reliable correlation between the variables, it is necessary to consider the level of sensitivity and specificity to determine the level of confidence of this tool to recognize and assess pain. Likewise, this could improve the prospects for choosing the thermal windows to be used in the species, as observed in this study, where the nasal thermal window did not present differences. This could be due to the presence of hair that would hinder heat radiation and its reading using IRT [16], which would reflect some of the limitations present in this study.

Finally, a limitation identified in this study was the lack of use of an assessment scale allowing for the recognition of acute pain in this species, as mentioned in similar models previously [12]. Another understandable limitation is the use of IRT in elective surgeries for healthy patients. Therefore, controlling these variables could benefit this technique itself. Hence, it would be essential to identify if this tool is applicable for recognizing acute pain in other clinical conditions in animals experiencing acute pain from different sources, such as skeletal muscle pain or neuropathic pain. Another limitation and potential area for future study would be to evaluate the ability of this technique to recognize the point where preoperative analgesia strategies are ineffective, thus indicating the need for rescue analgesia, as observed with thermal response in rehabilitation techniques [15]. Similarly, the present study considered only a single evaluator to record all the parameters. This might be a limitation since inter-observer reliability can influence pain evaluation [82,83]. Thus, future studies must consider different evaluators to assess this effect. On the other hand, the lack of consideration of other parameters, such as blood pressure, is another limitation of the study, as this parameter has a direct relationship with cardiovascular changes. Due to the difficulty of IRT application, developing a nociceptive thermographic index that facilitates its use in daily clinical practice could be valuable. Similarly, evaluation in a different surgical model involving stimuli in other tissues, such as bone or skeletal muscle, where a more intense stimulus occurs, could confirm whether CBD can control nociceptive stimuli [43].

## 5. Conclusions

In conclusion, using analgesics allows for controlling this autonomic response, which can be reflected in the facial thermal response. Based on this reasoning, the use of CBD alone or in combination with meloxicam controlled the nociceptive autonomic hemodynamic response measured through facial thermal response.

## Figures and Tables

**Table 1:** Temperature values of the different thermal windows (Mean ± SE) at the evaluation events (E) of 64 female dogs undergoing elective ovariohysterectomy surgery distributed into 4 study groups: G1, G2, G3 and G4.

Parameters	Treatments	Post-Surgical Events										***p* Value**
		E_Basal_	E_30min_	E_1h_	E_2h_	E_3h_	E_4h_	E_8h_	E_12h_	E_24h_	**E_48h_**	
OHT	G1 *n =* 16	36.7 ^1,a^ ± 0.22	35.9 ^1,a^ ± 0.35	36.0 ^1,a^ ± 0.21	35.6 ^1,a^ ± 0.37	36.4 ^1,a^ ± 0.20	35.7 ^1,a^ ± 0.37	36.5 ^1,a^ ± 0.32	36.0 ^1,a^ ± 0.26	36.2 ^1,a^ ± 0.14	36.2 ^1,a^ ± 0.35	*p =* 0.99
	G2 *n =* 16	37.2 ^1,a^ ± 0.19	36.5 ^1,a^ ± 0.34	37.2 ^1,a^ ± 0.21	37.5 ^2,a^ ± 0.16	36.8 ^1,a^ ± 0.37	37.2 ^1,a^ ± 0.34	37.3 ^1,a^ ± 0.23	36.3 ^1,a^ ± 0.27	36.9 ^1,a^ ± 0.24	37.1 ^1,a^ ± 0.30	*p =* 0.99
	G3 *n =* 16	37.4 ^1,a^ ± 0.16	37.2 ^1,a^ ± 0.21	37.3 ^1,a^ ± 0.22	37.1 ^1,2,a^ ± 0.19	37.1 ^1,a^ ± 0.24	36.4 ^1,a^ ± 0.32	37.3 ^1,a^ ± 0.17	36.6 ^1,a^ ± 0.26	36.7 ^1,a^ ± 0.24	37.1 ^1,a^ ± 0.17	*p =* 0.99
	G4 *n =* 16	36.9 ^1,a^ ± 0.39	36.5 ^1,a^ ± 0.25	37.3 ^1,a^ ± 0.30	37.3 ^2,a^ ± 0.24	37.1 ^1,a^ ± 0.28	36.9 ^1,a^ ± 0.24	37.1 ^1,a^ ± 0.17	36.7 ^1,a^ ± 0.21	37.0 ^1,a^ ± 0.18	37.4 ^1,a^ ± 0.14	*p =* 0.99
	*p* value	*p =* 0.98	*p =* 0.99	*p =* 0.99	***p =* 0.005**	*p =* 0.99	*p =* 0.99	*p =* 0.99	*p =* 0.99	*p =* 0.99	*p =* 0.89	
OLT	G1 *n =* 16	32.7 ^1,a^ ± 0.89	31.9 ^1,a^ ± 1.00	32.9 ^1,a^ ± 0.64	32.9 ^1,a^ ± 0.60	31.4 ^1,a^ ± 0.91	32.2 ^1,a^ ± 0.60	32.6 ^1,a^ ± 0.77	32.0 ^1,a^ ± 0.80	32.6 ^1,a^ ± 0.66	32.1 ^1,a^ ± 0.73	*p =* 0.89
	G2 *n =* 16	34.5 ^1,a^ ± 0.46	34.6 ^2,a^ ± 0.37	34.8 ^1,a^ ± 0.36	34.8 ^2,a^ ± 0.29	34.6 ^2,a^ ± 0.40	34.5 ^1,a^ ± 0.46	34.7 ^1,a^ ± 0.35	34.0 ^1,a^ ± 0.37	34.0 ^1,a^ ± 0.54	34.1 ^1,a^ ± 0.40	*p =* 0.99
	G3 *n =* 16	34.1 ^1,a^ ± 0.48	34.8 ^2,a^ ± 0.38	34.8 ^1,a^ ± 0.40	34.4 ^2,a^ ± 0.44	35.1 ^2,a^ ± 0.12	33.7 ^1,a^ ± 0.38	34.6 ^1,a^ ± 0.38	33.9 ^1,a^ ± 0.58	33.9 ^1,a^ ± 0.45	34.0 ^1,a^ ± 0.37	*p =* 0.94
	G4 *n =* 16	33.4 ^1,a^ ± 0.29	34.3 ^2,a^ ± 0.28	32.9 ^1,a^ ± 0.91	34.9 ^2,a^ ± 0.19	35.4 ^2,a^ ± 0.26	34.0 ^1,a^ ± 0.51	34.0 ^1,a^ ± 0.58	33.4 ^1,a^ ± 0.73	34.5 ^1,a^ ± 0.39	34.8 ^1,a^ ± 0.33	*p =* 0.78
	*p* value	*p =* 0.99	***p =* 0.05**	*p =* 0.93	***p =* 0.03**	***p =* 0.006**	*p =* 0.38	*p =* 0.42	*p =* 0.72	*p =* 0.68	*p =* 0.89	
UEHT	G1 *n =* 16	36.4 ^1,b^ ± 0.23	34.9 ^2,a^ ± 0.34	34.9 ^2,a^ ± 0.22	36.2 ^1,b^ ± 0.28	35.9 ^1,a,b^ ± 0.22	36.2 ^1,b^ ± 0.23	36.0 ^1,b^ ± 0.11	35.0 ^1,a,b^ ± 0.30	35.7 ^1,a,b^ ± 0.18	36.3 ^1,b^ ± 0.21	***p =* 0.007**
	G2 *n =* 16	36.7 ^1,a^ ± 0.18	36.0 ^1,2,a^ ± 0.30	36.5 ^1,a^ ± 0.23	36.6 ^1,a^ ± 0.20	36.0 ^1,a^ ± 0.35	36.8 ^1,a^ ± 0.32	36.7 ^1,a^ ± 0.22	35.7 ^1,a^ ± 0.26	35.9 ^1,a^ ± 0.28	36.2 ^1,a^ ± 0.22	*p =* 0.63
	G3 *n =* 16	37.0 ^1,a^ ± 0.18	36.6 ^1,a^ ± 0.32	36.6 ^1,a^ ± 0.28	36.6 ^1,a^ ± 0.21	36.7 ^1,a^ ± 0.15	36.6 ^1,a^ ± 0.23	37.0 ^1,a^ ± 0.14	35.9 ^1,b^ ± 0.31	35.7 ^1,b^ ± 0.18	36.3 ^1,a^ ± 0.24	***p =* 0.02**
	G4 *n =* 16	36.87 ^1,a^ ± 0.17	35.6 ^1,2,a^ ± 0.25	36.6 ^1,a^ ± 0.29	36.3 ^1,a^ ± 0.21	36.6 ^1,a^ ± 0.19	36.8 ^1,a^ ± 0.23	36.9 ^1,a^ ± 0.13	36.0 ^1,a^ ± 0.20	36.1 ^1,a^ ± 0.17	37.0 ^1,a^ ± 0.14	*p =* 0.99
	*p* value	*p =* 0.99	***p =* 0.0006**	***p =* 0.002**	*p =* 0.99	*p =* 0.99	*p =* 0.73	*p =* 0.99	*p =* 0.99	*p =* 0.32	*p =* 0.57	
UELT	G1 *n =* 16	35.0 ^1,b^ ± 0.42	32.1 ^1,a^ ± 0.33	33.8 ^1,a^ ± 0.26	32.9 ^1,a^ ± 0.35	32.3 ^1,a^ ± 1.10	33.9 ^1,a^ ± 0.35	34.0 ^1,a^ ± 0.47	31.8 ^1,a^ ± 0.84	33.9 ^1,a^ ± 0.32	34.4 ^1,a^ ± 0.29	***p =* 0.01**
	G2 *n =* 16	35.5 ^1,a^ ± 0.32	35.3 ^2,a^ ± 0.32	35.6 ^2,a^ ± 0.35	35.1 ^2,a^ ± 0.37	34.5 ^2,a^ ± 0.30	35.3 ^2,a^ ± 0.42	34.8 ^1,a^ ± 0.38	34.0 ^2,a^ ± 0.30	34.6 ^1,a^ ± 0.42	34.6 ^1,a^ ± 0.24	*p =* 0.99
	G3 *n =* 16	35.5 ^1,a^ ± 0.40	35.4 ^2,a^ ± 0.38	35.4 ^2,a^ ± 0.27	35.4 ^2,a^ ± 0.22	35.6 ^2,a^ ± 0.19	34.4 ^1,2,a^ ± 0.29	35.5 ^1,a^ ± 0.25	34.6 ^2,a^ ± 0.41	34.5 ^1,a^ ± 0.16	35.0 ^1,a^ ± 0.32	*p =* 0.99
	G4 *n =* 16	35.3 ^1,a^ ± 0.32	34.2 ^2,a^ ± 0.24	34.5 ^2,a^ ± 0.24	35.1 ^2,a^ ± 0.27	35.6 ^2,a^ ± 0.24	35.3 ^2,a^ ± 0.28	35.4 ^1,a^ ± 0.26	34.7 ^2,a^ ± 0.30	34.7 ^1,a^ ± 0.23	35.6 ^1,a^ ± 0.20	*p =* 0.99
	*p* value	*p =* 0.99	***p =* 0.0001**	***p =* 0.05**	***p =* 0.003**	***p =* 0.003**	***p =* 0.05**	*p =* 0.63	***p =* 0.01**	*p =* 0.99	*p =* 0.88	
LEHT	G1 *n =* 16	36.2 ^1,a^ ± 0.29	34.7 ^1,b^ ± 0.33	35.5 ^1,b^ ± 0.20	35.5 ^1,b^ ± 0.24	35.7 ^1,a,b^ ± 0.31	35.7 ^1,a,b^ ± 0.27	35.8 ^1,a,b^ ± 0.30	35.3 ^1,b^ ± 0.40	35.7 ^1,a,b^ ± 0.19	35.7 ^1,a,b^ ± 0.19	***p =* 0.05**
	G2 *n =* 16	36.8 ^1,a^ ± 0.22	36.0 ^2,a^ ± 0.30	37.0 ^2,a^ ± 0.27	37.0 ^1,a^ ± 0.16	36.6 ^1,a^ ± 0.21	37.2 ^2,a^ ± 0.26	36.6 ^1,a^ ± 0.23	35.8 ^1,a^ ± 0.29	35.9 ^1,a^ ± 0.41	36.2 ^1,a^ ± 0.41	*p =* 0.95
	G3 *n =* 16	37.1 ^1,a^ ± 0.21	36.7 ^2,a^ ± 0.24	37.0 ^2,a^ ± 0.20	36.6 ^1,a^ ± 0.23	36.7 ^1,a^ ± 0.18	36.7 ^1,2,a^ ± 0.21	36.8 ^1,a^ ± 0.12	36.0 ^1,a^ ± 0.33	35.8 ^1,a^ ± 0.22	36.4 ^1,a^ ± 0.24	*p =* 0.88
	G4 *n =* 16	37.1 ^1,b^ ± 0.23	35.9 ^2,b^ ± 0.19	36.1 ^1,b^ ± 0.24	36.9 ^1,a^ ± 0.22	36.7 ^1,a^ ± 0.18	36.5 ^1,2,a^ ± 0.22	36.7 ^1,a^ ± 0.19	36.1 ^1,a^ ± 0.17	36.2 ^1,a^ ± 0.27	36.7 ^1,a^ ± 0.27	*p =* 0.99
	*p* value	*p =* 0.99	***p =* 0.004**	***p =* 0.05**	*p =* 0.14	*p =* 0.81	*p =* 0.5	*p =* 0.61	*p =* 0.95	*p =* 0.95	*p =* 0.65	
LELT	G1 *n =* 16	34.6 ^1,a^ ± 0.37	33.3 ^1,a^ ± 0.42	34.0 ^1,a^ ± 0.36	33.9 ^1,a^ ± 0.36	34.4 ^1,a^ ± 0.62	34.2 ^1,a^ ± 0.34	34.3 ^1,a^ ± 0.41	33.4 ^1,a^ ± 0.52	34.5 ^1,a^ ± 0.30	33.0 ^1,a^ ± 0.59	*p =* 0.99
	G2 *n =* 16	35.6 ^1,a^ ± 0.31	35.8 ^2,a^ ± 0.32	36.2 ^2,a^ ± 0.26	35.9 ^2,a^ ± 0.29	35.9 ^2,a^ ± 0.28	36.5 ^2,a^ ± 0.31	36.2 ^2,a^ ± 0.22	35.3 ^2,a^ ± 0.26	34.9 ^1,a^ ± 0.55	35.4 ^2,a^ ± 0.30	*p =* 0.99
	G3 *n =* 16	35.5 ^1,a^ ± 0.29	35.8 ^2,a^ ± 0.34	36.5 ^2,a^ ± 0.20	35.8 ^2,a^ ± 0.21	36.0 ^2,a^ ± 0.14	36.0 ^1,2,a^ ± 0.22	35.9 ^2,a^ ± 0.14	35.6 ^2,a^ ± 0.13	34.9 ^1,a^ ± 0.26	35.2 ^2,a^ ± 0.33	*p =* 0.99
	G4 *n =* 16	35.3 ^1,a^ ± 0.48	35.4 ^2,a^ ± 0.19	35.5 ^1,2,a^ ± 0.25	35.6 ^2,a^ ± 0.27	36.1 ^2,a^ ± 0.17	36.0 ^1,2,a^ ± 0.19	36.2 ^2,a^ ± 0.11	35.8 ^2,a^ ± 0.21	35.8 ^1,a^ ± 0.16	35.9 ^2,a^ ± 0.21	*p =* 0.99
	*p* value	*p =* 0.99	***p =* 0.004**	***p =* 0.002**	***p =* 0.02**	***p =* 0.05**	***p =* 0.01**	***p =* 0.03**	***p =* 0.01**	*p =* 0.90	***p =* 0.009**	
NHT	G1 *n =* 16	31.9 ^1,a^ ± 0.79	29.3 ^1,a^ ± 0.59	30.1 ^1,a^ ± 0.88	31.6 ^1,a^ ± 0.68	31.9 ^1,a^ ± 0.80	31.5 ^1,a^ ± 0.79	31.6 ^1,a^ ± 0.93	30.7 ^1,a^ ± 0.75	30.6 ^1,a^ ± 0.57	31.9 ^1,a^ ± 0.63	*p =* 0.99
	G2 *n =* 16	33.6 ^1,a^ ± 0.61	32.7 ^1,a^ ± 0.93	33.4 ^1,a^ ± 1.03	33.3 ^1,a^ ± 0.91	33.3 ^1,a^ ± 0.90	33.8 ^1,a^ ± 1.06	33.3 ^1,a^ ± 0.86	30.8 ^1,a^ ± 1.08	32.0 ^1,a^ ± 0.81	32.4 ^1,a^ ± 0.82	*p =* 0.99
	G3 *n =* 16	33.7 ^1,a^ ± 0.83	32.4 ^1,a^ ± 1.14	33.0 ^1,a^ ± 0.92	33.1 ^1,a^ ± 1.00	33.5 ^1,a^ ± 0.87	32.4 ^1,a^ ± 0.86	33.7 ^1,a^ ± 0.83	31.5 ^1,a^ ± 1.10	32.1 ^1,a^ ± 0.78	32.3 ^1,a^ ± 0.77	*p =* 0.99
	G4 *n =* 16	33.3 ^1,b^ ± 1.04	30.8 ^1,a^ ± 0.92	31.1 ^1,a,b^ ± 1.06	32.2 ^1,b^ ± 1.22	32.2 ^1,b^ ± 1.08	32.6 ^1,b^ ± 0.90	33.4 ^1,b^ ± 0.90	30.3 ^1,a^ ± 0.83	31.1 ^1,a,b^ ± 0.90	32.8 ^1,b^ ± 0.95	***p =* 0.05**
	*p* value	*p =* 0.99	*p =* 0.99	*p =* 0.99	*p =* 0.99	*p =* 0.99	*p =* 0.99	*p =* 0.99	*p =* 0.99	*p =* 0.99	*p =* 0.99	
NMT	G1 *n =* 16	25.9 ^1,a^ ± 1.05	26.0 ^1,a^ ± 1.20	25.4 ^1,a^ ± 1.23	26.6 ^1,a^ ± 1.03	26.5 ^1,a^ ± 1.06	26.0 ^1,a^ ± 1.11	26.7 ^1,a^ ± 1.11	25.1 ^1,a^ ± 0.76	25.4 ^1,a^ ± 0.56	26.7 ^1,a^ ± 1.02	*p =* 0.99
	G2 *n =* 16	27.1 ^1,a^ ± 0.92	26.4 ^1,a^ ± 1.31	28.8 ^1,a^ ± 1.50	28.0 ^1,a^ ± 1.27	28.4 ^1,a^ ± 1.30	29.1 ^1,a^ ± 1.29	28.8 ^1,a^ ± 1.30	26.0 ^1,a^ ± 1.41	25.7 ^1,a^ ± 1.17	25.3 ^1,a^ ± 0.92	*p =* 0.99
	G3 *n =* 16	27.7 ^1,a^ ± 1.06	28.7 ^1,a^ ± 1.60	29.4 ^1,a^ ± 1.29	29.3 ^1,a^ ± 1.37	29.9 ^1,a^ ± 1.32	29.1 ^1,a^ ± 1.15	30.0 ^1,a^ ± 1.27	27.6 ^1,a^ ± 1.44	27.7 ^1,a^ ± 1.13	26.9 ^1,a^ ± 1.03	*p =* 0.99
	G4 *n =* 16	27.8 ^1,a^ ± 1.14	26.8 ^1,a^ ± 1.05	27.9 ^1,a^ ± 1.35	28.3 ^1,a^ ± 1.53	28.8 ^1,a^ ± 1.17	28.3 ^1,a^ ± 1.18	29.1 ^1,a^ ± 1.11	25.5 ^1,a^ ± 0.74	26.4 ^1,a^ ± 1.18	26.7 ^1,a^ ± 1.16	*p =* 0.99
	*p* value	*p =* 0.99	*p =* 0.99	*p =* 0.99	*p =* 0.99	*p =* 0.99	*p =* 0.99	*p =* 0.99	*p =* 0.99	*p =* 0.99	*p =* 0.99	
NLT	G1 *n =* 16	22.4 ^1,a^ ± 0.97	23.2 ^1,a^ ± 1.27	22.1 ^1,a^ ± 1.00	23.1 ^1,a^ ± 0.91	23.0 ^1,a^ ± 0.99	22.6 ^1,a^ ± 1.02	23.2 ^1,a^ ± 1.10	21.3 ^1,a^ ± 0.54	22.3 ^1,a^ ± 0.58	23.4 ^1,a^ ± 1.30	*p =* 0.99
	G2 *n =* 16	23.0 ^1,a^ ± 0.75	23.2 ^1,a^ ± 1.43	25.2 ^1,a^ ± 1.57	23.9 ^1,a^ ± 1.27	24.6 ^1,a^ ± 1.27	25.4 ^1,a^ ± 1.20	25.1 ^1,a^ ± 1.21	22.1 ^1,a^ ± 1.25	22.0 ^1,a^ ± 1.10	22.0 ^1,a^ ± 0.93	*p =* 0.99
	G3 *n =* 16	24.0 ^1,a^ ± 1.09	26.2 ^1,a^ ± 1.86	26.3 ^1,a^ ± 1.48	26.0 ^1,a^ ± 1.33	26.3 ^1,a^ ± 1.20	25.5 ^1,a^ ± 1.10	26.6 ^1,a^ ± 1.25	24.4 ^1,a^ ± 1.43	24.4 ^1,a^ ± 1.12	23.7 ^1,a^ ± 1.04	*p =* 0.99
	G4 *n =* 16	24.2 ^1,a^ ± 1.12	23.9 ^1,a^ ± 1.16	24.7 ^1,a^ ± 1.29	25.1 ^1,a^ ± 1.55	24.8 ^1,a^ ± 1.20	25.0 ^1,a^ ± 1.21	26.5 ^1,a^ ± 1.38	22.5 ^1,a^ ± 0.65	23.5 ^1,a^ ± 1.10	23.3 ^1,a^ ± 1.28	*p =* 0.99
	*p* value	*p =* 0.99	*p =* 0.99	*p =* 0.99	*p =* 0.99	*p =* 0.99	*p =* 0.99	*p =* 0.99	*p =* 0.99	*p =* 0.99	*p =* 0.99	

^a,b^: Different literals by row indicate significant differences between events for the same treatment. ^1,2^: Different numerals by column indicate significant differences between treatments for the same event. T = treatments (G1: negative group; G2: meloxicam group; G3: cannabidiol group; G4: cannabidiol + meloxicam group). E: post-surgical events (E_Basal_: 30 min pre-surgery; E_30min_: 30 min post-surgery; E_1h_: 1 h post-surgery; E_2h_: 2 h post-surgery; E_3h_: 3 h post-surgery; E_4h_: 4 h post-surgery; E_8h_: 8 h post-surgery; E_12h_: 12 h post-surgery, E_24h_: 24 h post-surgery; E_48h_: 48 h post-surgery). OHT: Ocular high temperature. OLT: Ocular low temperature. UEHT: Upper eyelid high temperature. UELT: Upper eyelid low temperature. LEHT: Lower eyelid high temperature. LELT: Lower eyelid low temperature. LETG: Left eye tear gland. NHT: Nasal high temperature. NMT: Nasal mean temperature. NLT: Nasal low temperature.

**Table 2:** Physiological parameters (Mean ± SE) at evaluation events (E) of 64 female dogs undergoing elective ovariohysterectomy surgery distributed into 4 study groups: G1, G2, G3 and G4.

Parameters	Treatments	Post-Surgical Events										*p* Value
		E_Basal_	E_30min_	E_1h_	E_2h_	E_3h_	E_4h_	E_8h_	E_12h_	E_24h_	E_48h_	
HR	G1 *n* = 16	116 ^1,a^ ± 11	103 ^1,a^ ± 4	119 ^1,a^ ± 8	124 ^1,a^ ± 9	110 ^1,a^ ± 8	112 ^1,a^ ± 11	101 ^1,a^ ± 11	101 ^1,a^ ± 5	100 ^1,a^ ± 5	101 ^1,a^ ± 5	*p =* 0.99
	G2 *n =* 16	129 ^1,a^ ± 5	108 ^1,b^ ± 7	104 ^2,b^ ± 5	108 ^2,b^ ± 4	114 ^1,b^ ± 6	105 ^1,b^ ± 7	97 ^1,b^ ± 4	100 ^1,b^ ± 2	108 ^1,b^ ± 7	97 ^1,b^ ± 4	***p =* 0.02**
	G3 *n =* 16	120 ^1,a^ ± 8	105 ^1,a^ ± 6	110 ^2,a^ ± 3	108 ^2,a^ ± 4	104 ^1,a^ ± 6	103 ^1,a^ ± 6	102 ^1,a^ ± 4	101 ^1,a^ ± 4	102 ^1,a^ ± 4	96 ^1,a^ ± 5	*p =* 0.99
	G4 *n* = 16	121 ^1,a^ ± 6	104 ^1,a^ ± 6	108 ^2,a^ ± 6	107 ^2,a^ ± 4	104 ^1,a^ ± 4	101 ^1,a^ ± 5	106 ^1,a^ ± 4	101 ^1,a^ ± 3	107 ^1,a^ ± 5	105 ^1,a^ ± 4	*p =* 0.99
	*p* value	*p =* 0.98	*p =* 0.99	***p =* 0.05**	***p =* 0.05**	*p =* 0.99	*p =* 0.99	*p =* 0.99	*p =* 0.99	*p =* 0.99	*p =* 0.73	
RR	G1 *n* = 16	43 ^1,a^ ± 7	35 ^2,b^ ± 8	40 ^1,a,b^ ± 8	39 ^1,b^ ± 7	37 ^1,b^ ± 8	36 ^1,b^ ± 7	36 ^1,b^ ± 7	27 ^1,c^ ± 4	31 ^1,b,c^ ± 3	31 ^1,b,c^ ± 3	***p =* 0.03**
	G2 *n =* 16	44 ^1,a^ ± 4	40 ^1,a^ ± 4	31 ^2,b^ ± 3	32 ^2,b^ ± 2	35 ^1,b^ ± 3	32 ^1,b^ ± 4	29 ^2,c^ ± 3	26 ^1,c^ ± 2	30 ^1,b,c^ ± 4	34 ^1,b,c^ ± 4	***p =* 0.03**
	G3 *n =* 16	39 ^1,a^ ± 4	29 ^3,b^ ± 2	30 ^2,b^ ± 2	29 ^2,b^ ± 2	28 ^1,b^ ± 2	31 ^1,b^ ± 1	26 ^2,b^ ± 1	25 ^1,b^ ± 3	25 ^1,b^ ± 2	31 ^1,b^ ± 3	***p =* 0.05**
	G4 *n* = 16	45 ^1,a^ ± 4	36 ^2,b^ ± 2	31 ^2,b^ ± 2	28 ^2,b^ ± 2	30 ^1,b^ ± 2	30 ^1,b^ ± 2	27 ^2,b^ ± 2	28 ^1,b^ ± 2	27 ^1,b^ ± 2	29 ^1,b^ ± 2	***p =* 0.04**
	*p* value	*p =* 0.99	***p =* 0.05**	***p =* 0.05**	***p =* 0.02**	*p =* 0.51	*p =* 0.99	***p =* 0.01**	*p =* 0.99	*p =* 0.86	*p =* 0.99	
T ^a^ C	G1 *n* = 16	38.6 ^1,a^ ± 0.15	37.2 ^1,b^ ± 0.50	37.5 ^1,a,b^ ± 0.44	38.3 ^1,a,b^ ± 0.19	38.5 ^1,a^ ± 0.16	38.4 ^1,a^ ± 0.17	38.5 ^1,a^ ± 0.26	37.9 ^1,a,b^ ± 0.25	38.3 ^1,a^ ± 0.22	38.3 ^1,a^ ± 0.24	***p =* 0.006**
	G2 *n =* 16	38.6 ^1,a^ ± 0.17	37.5 ^2,b^ ± 0.21	38.0 ^1,a^ ± 0.14	38.2 ^2,a^ ± 0.11	38.2 ^2,a^ ± 0.13	38.2 ^1,a^ ± 0.09	38.2 ^1,a^ ± 0.10	38.1 ^1,a^ ± 0.13	38.2 ^1,a^ ± 0.12	38.2 ^1,a^ ± 0.09	***p =* 0.007**
	G3 *n =* 16	38.6 ^1,a^ ± 0.09	37.6 ^2,b^ ± 0.19	37.9 ^1,a,b^ ± 0.16	38.0 ^2,a,b^ ± 0.15	38.0 ^2,a,b^ ± 0.07	38.2 ^1,a,b^ ± 0.10	38.2 ^1,a,b^ ± 0.10	38.1 ^1,a,b^ ± 0.14	37.9 ^1,a,b^ ± 0.21	38.2 ^1,a,b^ ± 0.13	***p =* 0.001**
	G4 *n* = 16	38.5 ^1,a^ ± 0.10	37.4 ^2,b^ ± 0.17	37.6 ^1,b^ ± 0.20	37.9 ^2,a,b^ ± 0.12	37.7 ^2,a,b^ ± 0.21	37.8 ^1,a,b^ ± 0.12	38.1 ^1,a,b^ ± 0.11	38.1 ^1,a,b^ 0.10	38.1 ^1,a,b^ ± 0.17	38.4 ^1,a^ ± 0.12	***p =* 0.01**
	*p* value	*p =* 0.99	*p =* 0.99	*p =* 0.93	*p =* 0.99	*p =* 0.99	*p =* 0.38	*p =* 0.42	*p =* 0.72	*p =* 0.68	*p =* 0.89	

^a,b,c^: Different literals by row indicate significant differences between events for the same treatment. ^1,2,3^: Different numerals by column indicate significant differences between treatments for the same event. T = treatments (G1: negative group; G2: meloxicam group; G3: cannabidiol group; G4: cannabidiol + meloxicam group). E: Post-surgical events (E_Basal_: 30 min pre-surgery; E_30min_: 30 min post-surgery; E_1h_: 1 h post-surgery; E_2h_: 2 h post-surgery; E_3h_: 3 h post-surgery; E_4h_: 4 h post-surgery; E_8h_: 8 h post-surgery; E_12h_: 12 h post-surgery, E_24h._: 24 h post-surgery; E_48h._: 48 h post-surgery). HR: Heart Rate. RR: Respiratory rate. T °C: Rectal temperature.

**Table 3:** Correlation between the superficial temperature of different thermal windows and physiological parameters.

r *p* Value	HR	RR	T °C	OHT	OMT	OLT	LETG	UEHT	UEMT	UELT	LEHT	LEMT	LELT	NHT	NMT	NLT
FC	1.000															
FR	0.194 0.085	1.000														
T °C	0.165 0.172	0.091 0.455	1.000													
OHT	0.037 0.749	0.123 0.282	−0.005 0.967	1.000												
OMT	0.040 0.727	0.091 0.429	−0.059 0.632	0.785 0.000	1.000											
OLT	−0.103 0.371	0.033 0.774	−0.033 0.791	0.496 0.000	0.722 0.000	1.000										
LETG	0.041 0.718	0.015 0.897	−0.047 0.697	0.753 0.000	0.773 0.000	0.649 0.000	1.000									
UEHT	−0.100 0.375	0.011 0.920	0.042 0.729	0.732 0.000	0.771 0.000	0.461 0.000	0.697 0.000	1.000								
UEMT	−0.050 0.657	−0.026 0.821	−0.005 0.970	0.541 0.000	0.667 0.000	0.457 0.000	0.610 0.000	0.758 0.000	1.000							
UELT	−0.004 0.970	−0.005 0.962	−0.082 0.499	0.439 0.000	0.628 0.000	0.573 0.000	0.584 0.000	0.633 0.000	0.670 0.000	1.000						
LEHT	0.063 0.580	0.030 0.791	0.014 0.906	0.773 0.000	0.866 0.000	0.562 0.000	0.762 0.000	0.874 0.000	0.698 0.000	0.617 0.000	1.000					
LEMT	0.040 0.725	0.042 0.713	−0.075 0.536	0.735 0.000	0.861 0.000	0.611 0.000	0.762 0.000	0.835 0.000	0.691 0.000	0.623 0.000	0.957 0.000	1.000				
LELT	−0.042 0.709	0.001 0.996	−0.140 0.249	0.594 0.000	0.774 0.000	0.631 0.000	0.692 0.000	0.714 0.000	0.615 0.000	0.597 0.000	0.814 0.000	0.914 0.000	1.000			
NHT	0.061 0.592	−0.004 0.975	−0.092 0.448	0.483 0.000	0.523 0.000	0.465 0.000	0.548 0.000	0.517 0.000	0.495 0.000	0.522 0.000	0.578 0.000	0.559 0.000	0.500 0.000	1.000		
NMT	0.067 0.552	0.010 0.927	−0.049 0.690	0.446 0.000	0.498 0.000	0.411 0.000	0.436 0.000	0.475 0.000	0.478 0.000	0.452 0.000	0.548 0.000	0.526 0.000	0.468 0.000	0.868 0.000	1.000	
NLT	0.058 0.608	−0.013 0.911	−0.049 0.686	0.346 0.000	0.384 0.000	0.340 0.000	0.335 0.000	0.367 0.000	0.388 0.000	0.349 0.000	0.440 0.000	0.423 0.000	0.376 0.000	0.730 0.000	0.930 0.000	1.000

HR: Heart Rate. RR: Respiratory rate. T °C: Rectal temperature. OHT: Ocular high temperature. OMT: Ocular medium temperature. OLT: Ocular low temperature. UEHT: Upper eyelid high temperature. UEMT: Upper eyelid medium temperature. UELT: Upper eyelid low temperature. LEHT: Lower eyelid high temperature. LEMT: Lower eyelid medium temperature. LELT: Lower eyelid low temperature. LETG: Left eye tear gland. NHT: Nasal high temperature. NMT: Nasal medium temperature. NLT: Nasal low temperature.

## Data Availability

Data are contained within the article.
